# Gemtuzumab ozogamicin plus standard induction chemotherapy improves outcomes of newly diagnosed intermediate cytogenetic risk acute myeloid leukemia

**DOI:** 10.1038/s41408-023-00910-x

**Published:** 2023-09-04

**Authors:** Hassan Awada, Mina Abdelmalek, Tara Cronin, Jeffrey Baron, Zakariya Kashour, Farhan Azad, Muhammad Salman Faisal, Mark Faber, Matthew Gravina, Pamela J. Sung, Steven D. Green, Amanda Przespolewski, James E. Thompson, Elizabeth A. Griffiths, Eunice S. Wang

**Affiliations:** 1 Leukemia Service, Department of Medicine, Roswell Park Comprehensive Cancer Center, Buffalo, NY USA; 2 Department of Medicine, State University of New York at Buffalo, Buffalo, NY USA; 3 Department of Pharmacy, Roswell Park Comprehensive Cancer Center, Buffalo, NY USA; 4 Department of Pharmacology & Therapeutics, Roswell Park Comprehensive Cancer Center, Buffalo, NY USA

**Keywords:** Acute myeloid leukaemia, Cytogenetics, Cancer genomics

## To the Editor:

Gemtuzumab ozogamicin (GO) is a CD33-directed antibody-drug conjugate approved for the treatment of newly diagnosed (ND) and refractory/relapsed CD33-positive acute myeloid leukemia (AML) [1–6]. A meta-analysis of individual patient data from 5 randomized controlled trials (*n* = 3325) confirmed a significant survival benefit of GO added to intensive induction chemotherapy for patients with AML without adverse cytogenetics [7]. While patients with favorable cytogenetic ND-AML, specifically core-binding factor AML (CBF-AML), particularly benefited with a 5-year overall survival improvement of 20.7% (log-rank *p* = 0.0006), patients with intermediate cytogenetic risk AML also showed a significant 5-year survival improvement of 5.7% (*p* = 0.005). The smaller survival difference in this latter patient group coupled with the apprehension for sinusoidal obstruction syndrome (SOS)/veno-occlusive disease (VOD), particularly following allogeneic hematopoietic stem cell transplantation (allo-HSCT), has led many clinicians to omit GO from intensive induction for patients with intermediate-risk AML [8]. To assess whether the addition of GO to intensive induction therapy is beneficial in this intermediate-risk patient subset, we performed a retrospective analysis of real-world outcomes of 7 + 3 plus GO as compared with 7 + 3 alone in patients with intermediate cytogenetic risk ND-AML treated at our comprehensive cancer center.

Demographic, clinical, and genomic information were collected from adult patients with ND intermediate cytogenetic risk AML who were treated with intensive chemotherapy at Roswell Park Comprehensive Cancer Center between 2015 and 2023. Risk stratification was defined per the European LeukemiaNet (ELN) 2022 classification [9]. Genomic profiling was performed using next-generation sequencing (Foundation One Heme). 7 + 3 plus GO patients received at least one dose of GO 3 mg/m^2^ (up to one 4.5 mg vial) plus cytarabine 100–200 mg/m^2^ continuous IV infusion on days 1–7, and daunorubicin 60–90 mg/m² IV on days 1–3 (7 + 3). Control patients received 7 + 3 alone. No patients received additional frontline therapy (e.g., FLT3 inhibitors). All patients who subsequently underwent allo-HSCT received prophylactic enoxaparin to prevent hepatic SOS before and/or following transplantation as an institutional standard if platelets were ≥ 50 ×10^9^/L. Allo-HSCT patients also received ursodiol prophylaxis per institutional protocol.

Study endpoints included response rates as defined per 2022 ELN criteria [9], measurable residual disease (MRD) as detected by multiparameter flow cytometry (MFC, sensitivity <0.01%), relapse rate, rate of successful bridging to allo-HSCT, leukemia-free survival, overall survival, and overall survival censored at time of allo-HSCT. Complete remission (CR) after induction treatment was defined as < 5% marrow blasts; absence of circulating blasts; absence of extramedullary disease; with an absolute neutrophil count (ANC) > 1 ×10^9^/L and platelets > 100 ×10^9^/L. Complete remission with incomplete count recovery (CRi) was defined as < 5% marrow blasts in the setting of an ANC < 1 × 10^9^/L and/or platelets < 100× 10^9^/L [10]. Additional information included time from the first day of induction therapy to neutrophil (ANC ≥ 1 × 10^9^/L) and platelet (≥100 × 10^9^/L) count recovery in patients who achieved CR/CRi and incidence of SOS/VOD. Patient characteristics were summarized as range and medians (for continuous variables) and counts as percentages (for categorical variables). Chi-square analysis was used to compare categorical data while Mann Whitney U test was used to compare continuous data, respectively. All *p* values were two-sided. Survival estimates were calculated by the Kaplan–Meier method, and survival curves were compared using log-rank statistics.

One hundred thirteen patients were evaluated with a median follow-up of 26.8 months (range: 1.3 - 102.7 months). Thirty-three (29%) received 7 + 3 plus GO and 80 received 7 + 3 (71%). All 33 patients in the 7 + 3 plus GO group received 4.5 mg of GO per dose with a cumulative number of doses of 3 (84%), 2 (13%), and 1 (3%). Six patients (18%) received consolidation with GO: 1 course (*n* = 2), 2 courses (*n* = 2), and 3 courses (*n* = 2). Baseline demographic, clinical and molecular characteristics of patients in both groups are summarized in Table 1. The median age of patients who received 7 + 3 plus GO (57 years; range: 19–73 years) and 7 + 3 (59 years; range: 22–87 years) was similar. Additionally, there was no significant difference in the percentage of patients ≥ 60 years in both groups (7 + 3 plus GO 45% vs 7 + 3 48%). Gender was equally distributed. Subtypes of AML including primary/de novo AML (pAML), secondary AML (sAML), and therapy-related AML (tAML) were statistically similar. When molecular profiles were compared, ELN 2022 molecular risk stratification groups with defined favorable, intermediate, and adverse molecular features were comparable between both treatment arms without significant difference in percentages (Table 1). However, patients in the 7 + 3 plus GO group were significantly enriched for mutations in *DNMT3A* (33 vs 5%, *p* = 0.0001) and *IDH 1/2* (36 vs 15%, *p* = 0.02), while patients treated with 7 + 3 had a significantly higher frequency of *NPM1* mutations (23 vs 3%, *p* = 0.01). Among those undergoing allo-HSCT, 80% of those in the 7 + 3 plus GO group received reduced-intensity conditioning (RIC) and 20% received myeloablative conditioning (MAC). These percentages were similar for patients in the 7 + 3 plus group (91% RIC and 9% MAC; *p* = 0.4).

**Table 1:** Patients’ baseline demographic, clinical and molecular features.

Group/Variables	7 + 3	7 + 3 plus GO	p-value
**n (%)**	80 (71)	33 (29)	
**Follow-up** (mo), median (range)	24.3 (4–62.0)	29.8 (1.3–102.7)	
**Accrual dates**, range (mo/yr)	1/2018–8/2021	1/2015–6/2022	
**Age** (y), median (range)	57 (19–73)	59 (22–87)	ns
≥60 y, *n* (%)	15 (45)	38 (48)	ns
Female/Male, n (%)	18 (55)/15 (45)	37 (46)/43 (54)	ns
**AML subtype**			ns
pAML, *n* (%)	68 (85)	30 (91)	ns
sAML, *n* (%)	8 (10)	2 (6)	ns
tAML, *n* (%)	4 (5)	1 (3)	ns
**Mortality**			
30-day, *n* (%)	1 (1.3)	0 (0)	ns
60-day, *n* (%)	3 (3.8)	0 (0)	ns
**Conditioning chemotherapy prior to allo-HSCT (%)**			
Myeloablative regimens	9	20	ns
Reduced-intensity regimens	91	80	ns
**Gene mutations (%)**			
*ASXL1*	14	6	ns
*DNMT3A*	5	33	0.0001
*FLT3 (ITD/TKD)*	11	12	ns
*IDH1/2*	15	36	0.02
*NPM1*	23	3	0.01
*KRAS/NRAS*	23	21	ns
*RUNX1*	14	9	ns
*TP53*	3	3	ns
**Molecular risk stratification per ELN 2022**			
Favorable, n (%)	15 (19)	8 (24)	ns
Intermediate, n (%)	29 (37)	10 (30)	ns
Adverse, n (%)	34 (44)	15 (46)	Ns

*n* number, *mo* month, *y* years, *ns* not statistically significant, *GO* gemtuzumab ozogamicin, *AML* acute myeloid leukemia, *pAML* primary/ de novo AML, *sAML* secondary AML, *tAML* therapy-related AML, *allo-HSCT* allogeneic hematopoietic stem cell transplantation, *ELN* European LeukemiaNet.

**Fig. 1:**
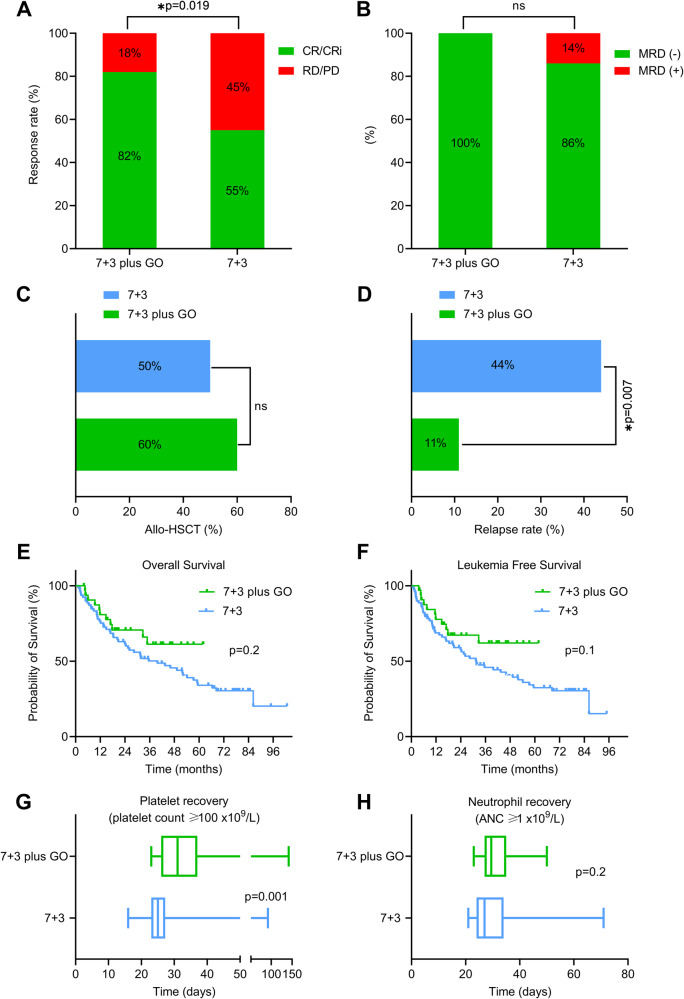
Comprehensive clinical responses and survival outcomes comparisons between 7 + 3 plus gemtuzumab ozogamicin (GO) vs 7 + 3 groups. Bar diagrams demonstrating the percentage of complete remission (CR) and complete remission with incomplete count recovery (CRi) response rates after intensive induction chemotherapy (**A**), measurable residual disease (MRD) status at time of CR/CRi (**B**), percentage of patients who underwent allogeneic stem cell transplantation (allo-HSCT) (**C**), and relapse rate after CR/CRi (**D**) in 7 + 3 plus GO vs 7 + 3 groups. Kaplan-Meier curves showing overall survival (**E**) and leukemia free survival (**F**) in 7 + 3 plus GO vs 7 + 3 groups. Scatter plots illustrating time (in days) to platelet (**G**) and neutrophil (**H**) recovery in CR/CRi patients who received 7 + 3 plus GO vs 7 + 3 regimen. ns not significant; ANC absolute neutrophil count.

Patients treated with 7 + 3 plus GO achieved significantly higher rates of CR/CRi (27/33, 82%) compared to those receiving 7 + 3 (44/80, 55%; *p* = 0.019; Fig. 1A). Among patients with available MRD status, 100% achieved MRD-negative CR/CRi after 7 + 3 plus GO (18/18) compared to 86% with 7 + 3 (12/14, *p* = 0.2; Fig. 1B). Among patients achieving CR/CRi, 60% (*n* = 16) after 7 + 3 plus GO vs 50% (*n* = 22) after 7 + 3 were successfully bridged to allo-HSCT (*p* = 0.5; Fig. 1C). The cumulative incidence of relapse after CR/CRi was significantly lower after 7 + 3 plus GO than 7 + 3 (11 vs 44%; *p* = 0.007; Fig. 1D). In addition, 7 + 3 plus GO treated patients demonstrated a superior, although not statistically significant, overall survival benefit (median not reached) compared to 7 + 3 alone (median 39 months; Fig. 1E). The survival trend was preserved when censored at the time of allo-HSCT (32.6 vs 16.9 months; Fig. S1). Moreover, leukemia-free survival (LFS) was numerically better in the 7 + 3 plus GO (median not reached) vs 7 + 3 group, although the improvement was not statistically significant (median 31.5 months, *p* = 0.1; Fig. 1F). Patients who received 7 + 3 plus GO had significantly longer median duration to platelet count recovery (31 vs 25 days, *p* = 0.001; Fig. 1G), while similar time to neutrophil recovery (29.5 vs 27 days, *p* = 0.2; Fig. 1H). No patients receiving 7 + 3 plus GO developed SOS/VOD.

We compared the molecular features of responders and nonresponders in each treatment group to identify potential molecular determinants of response. In the 7 + 3 + GO treatment group, nonresponding AML patients were enriched for mutations in *ASXL1, DNMT3A, TET2*, and *TP53* while responders were more likely to harbor *FLT3, IDH1/2, KRAS/NRAS* and *RUNX1* mutations (Fig. S2). In the 7 + 3 group, nonresponding AML patients were significantly enriched for mutations in *ASXL1* and *BCOR/BCORL1*, while *NPM1* mutations were more significantly common in responders (Fig. S3).

The results of our study reiterate the previously reported advantage of GO addition to cytarabine and anthracycline-based intensive induction chemotherapy in ND-AML with intermediate-risk cytogenetics [7]. This was reflected by significantly improved response rates and decreased risk of relapse following 7 + 3 plus GO. Although statistically significant prolonged overall survival was not achieved likely due to sample size and limited follow-up, the remarkable clinical advantage of GO was evident. This was further supported by the matched-group design, with balanced ELN 2022 molecular risk distribution, and similar toxicity profile except prolonged platelet count recovery with GO. Not surprisingly, molecular features appeared to be key determinants of response in both treatment groups, supporting the need for molecularly targeted approaches. Nonetheless, our data demonstrated improved clinical outcomes following 7 + 3 plus GO in the majority of intermediate cytogenetic risk AML patients. Interestingly, a recent phase 3 trial (AMLSG 09–09) in *NPM1* mutant AML patients demonstrated that GO added to intensive upfront chemotherapy decreased relapse risk and improved event-free survival but did not benefit *FLT3* co-mutated patients [11]. Based on this, we can argue that the significantly lower percentage of *NPM1* mutations in the 7 + 3 plus GO group compared to 7 + 3 group, might have affected the significance of outcome comparison between both groups. Of note, *FLT3* mutated patients included in our study were treated prior to the approval of midostaurin in 2017, after which all *FLT3* mutated patients received a *FLT3* targeted agent with induction chemotherapy. Although our center’s experience suggests that GO may be safely combined with *FLT3* inhibition and intensified anthracycline-based induction regimens in younger patients [12], this data is limited to a very few cases and therefore, larger clinical trials are necessary to unravel the feasibility and efficacy of this combinatorial approach. Promisingly, a recent study demonstrated high response rate and good tolerability with no evidence of increased toxicity of GO plus midostaurin added to standard intensive chemotherapy in patients with newly diagnosed *FLT3*-mutated/CD33 + AML, with exception of slightly prolonged recovery of neutrophils [13].

Study limitations include its small sample size, retrospective design, and influence of other factors such as mutation differences and physician selection on clinical outcomes of patients treated at a single academic center. Although patients in the 7 + 3 plus GO cohort were treated more recently than those receiving 7 + 3 alone, leukemia expertise and support measures did not differ substantially over these time frames as reflected by low mortality rates at 30- and 60-days after treatment initiation (Table 1). Therefore, the generalizability of these results to a larger more diverse cohort is not certain. Moreover, MRD was measured by MFC only and was available for only a limited number of patients.

In conclusion, newly diagnosed adult patients with intermediate cytogenetic risk AML treated with 7 + 3 plus GO at our academic center had higher rates of MRD-negative CR and CRi, lower rates of relapse, and a trend to improved overall survival and increased rate of subsequent allo-HSCT as compared with 7 + 3 alone. 7 + 3 plus GO was associated with prolonged thrombocytopenia but no SOS/VOD with subsequent allo-HSCT. Non-responders in the 7 + 3 plus GO group harbored adverse risk genetic mutations that likely dictated the failure to respond. Although the addition of GO was associated with a non-significant trend towards improved overall survival and LFS, larger samples and longer follow-up are warranted to decipher an absolute survival advantage of this regimen. Nevertheless, our results support the potential benefit and relative safety of adding GO to intensive chemotherapy for intermediate cytogenetic risk AML.

### Supplementary information

SUPPLEMENTAL MATERIAL
