# Supplementary material for: Gemtuzumab ozogamicin plus standard induction chemotherapy improves outcomes of newly diagnosed intermediate cytogenetic risk acute myeloid leukemia
Source: Blood Cancer J. 2023 Sep 4;13(1):131. doi: 10.1038/s41408-023-00910-x (PMC10477319; doi:10.1038/s41408-023-00910-x)
Supplement: Supplementary file 1 — SUPPLEMENTAL MATERIAL [file 41408_2023_910_MOESM1_ESM.docx]

**Supplemental Material**

**Title:** Gemtuzumab Ozogamicin plus Standard Induction Chemotherapy Improves Outcomes of Newly Diagnosed Intermediate Risk Cytogenetic Acute Myeloid Leukemia.

**Authors: Hassan Awada^1*^, Mina Abdelmalek^2*^, Tara Cronin^1^, Jeffrey Baron^3^, Zakariya Kashour^2^, Farhan Azad^2^, Mark Faber^1^, Matthew Gravina^1^, Pamela Sung^1^, Steven D. Green^1^, Amanda Przespolewski^1^, James E. Thompson^1^, Elizabeth A. Griffiths^1^ and Eunice S. Wang^1^**

**Affiliations:**

**^1^ Leukemia Service, Department of Medicine, Roswell Park Comprehensive Cancer Center, Buffalo, NY.**

**^2^ Department of Medicine, State University of New York at Buffalo, Buffalo, NY.**

**^3^ Department of Pharmacy, Roswell Park Comprehensive Cancer Center, Buffalo, NY.**

***These authors contributed equally**

**Content:**

**Supplemental Figure 1: Survival outcomes in 7+3 plus gemtuzumab ozogamicin (GO) vs 7+3 groups.**

**Supplemental Figure 2: Molecular determinants of response to 7+3 plus gemtuzumab ozogamicin (GO).**

**Supplemental Figure 3: Molecular determinants of response to 7+3.**

**Supplemental Figure 1**
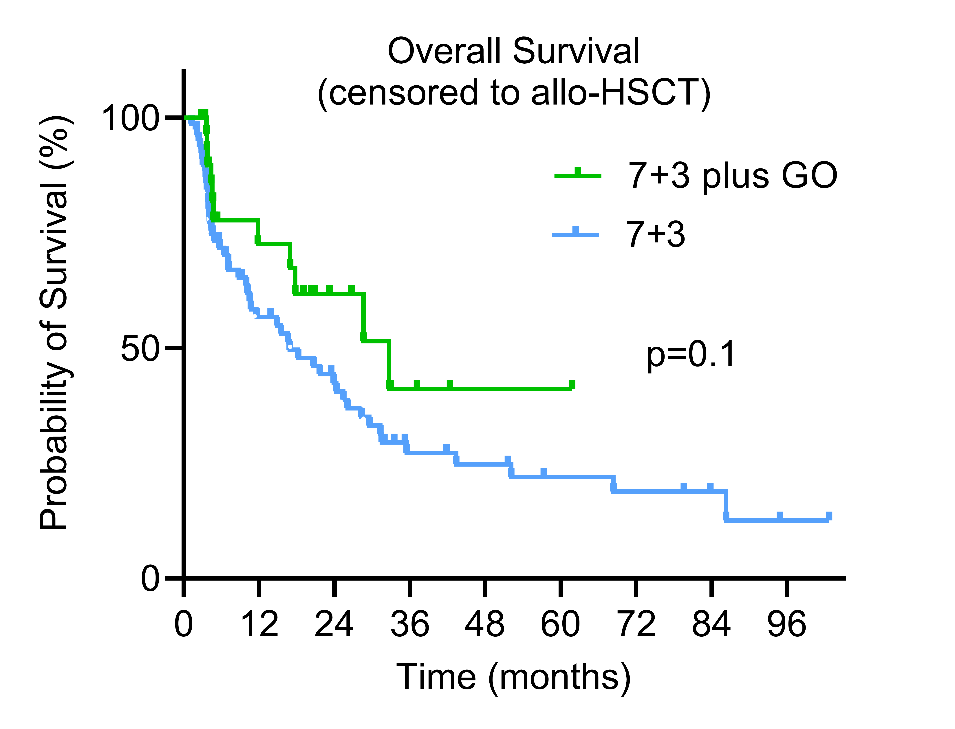


**Supplemental Figure 1: Survival outcomes in 7+3 plus gemtuzumab ozogamicin (GO) vs 7+3 groups.** Kaplan-Meier curves showing overall survival (in months) censored to allogeneic hematopoietic stem cell transplantation (allo-HSCT) in 7+3 plus GO vs 7+3 groups.

**Supplemental Figure 2**

**
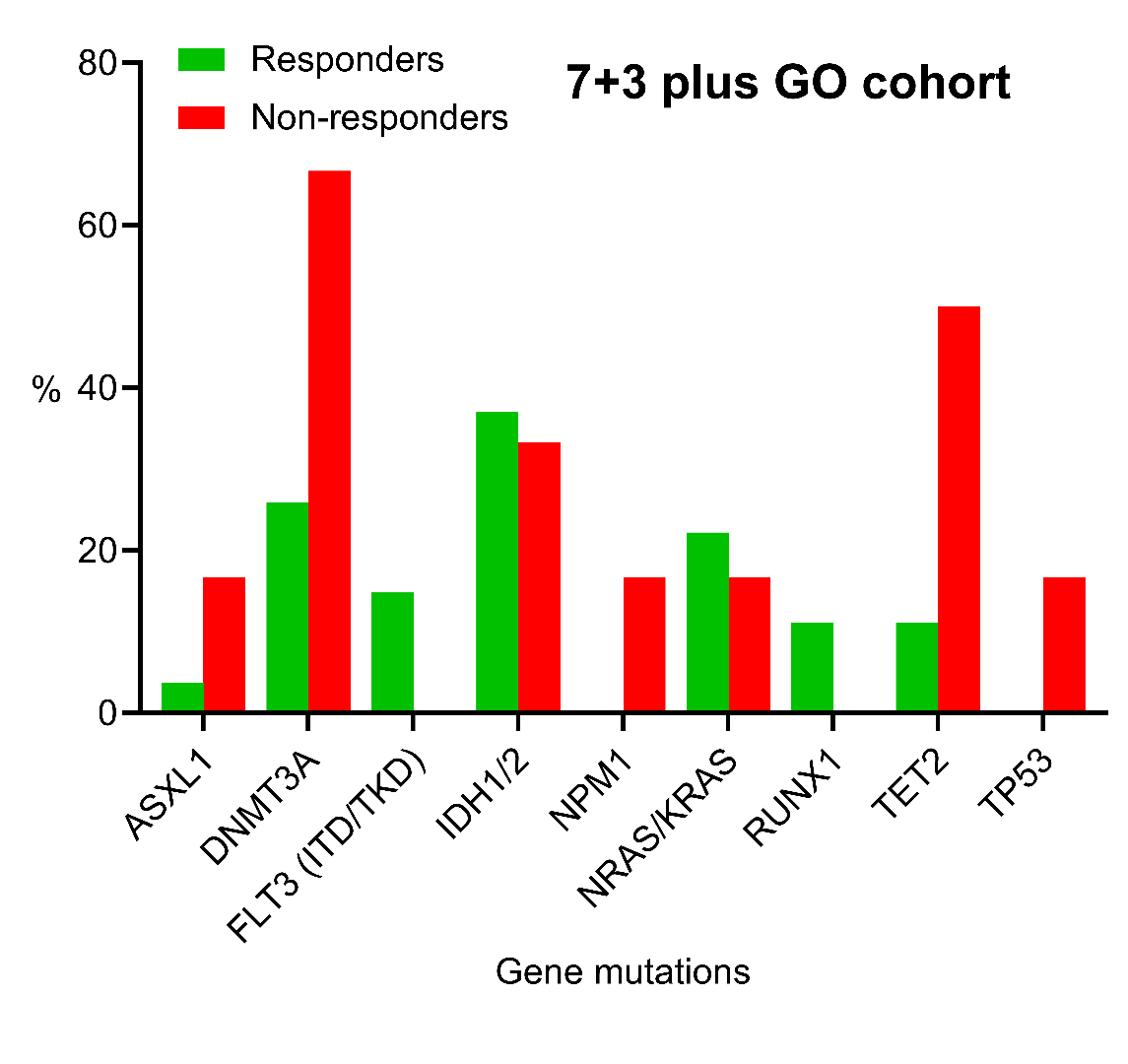
**

**Supplemental Figure 2: Molecular determinants of response to 7+3 plus gemtuzumab ozogamicin (GO) treatment.**  Bar diagrams showing the percentages of gene mutations detected in responder’s vs non-responders to 7+3 plus GO regimen.

**Supplemental Figure 3**


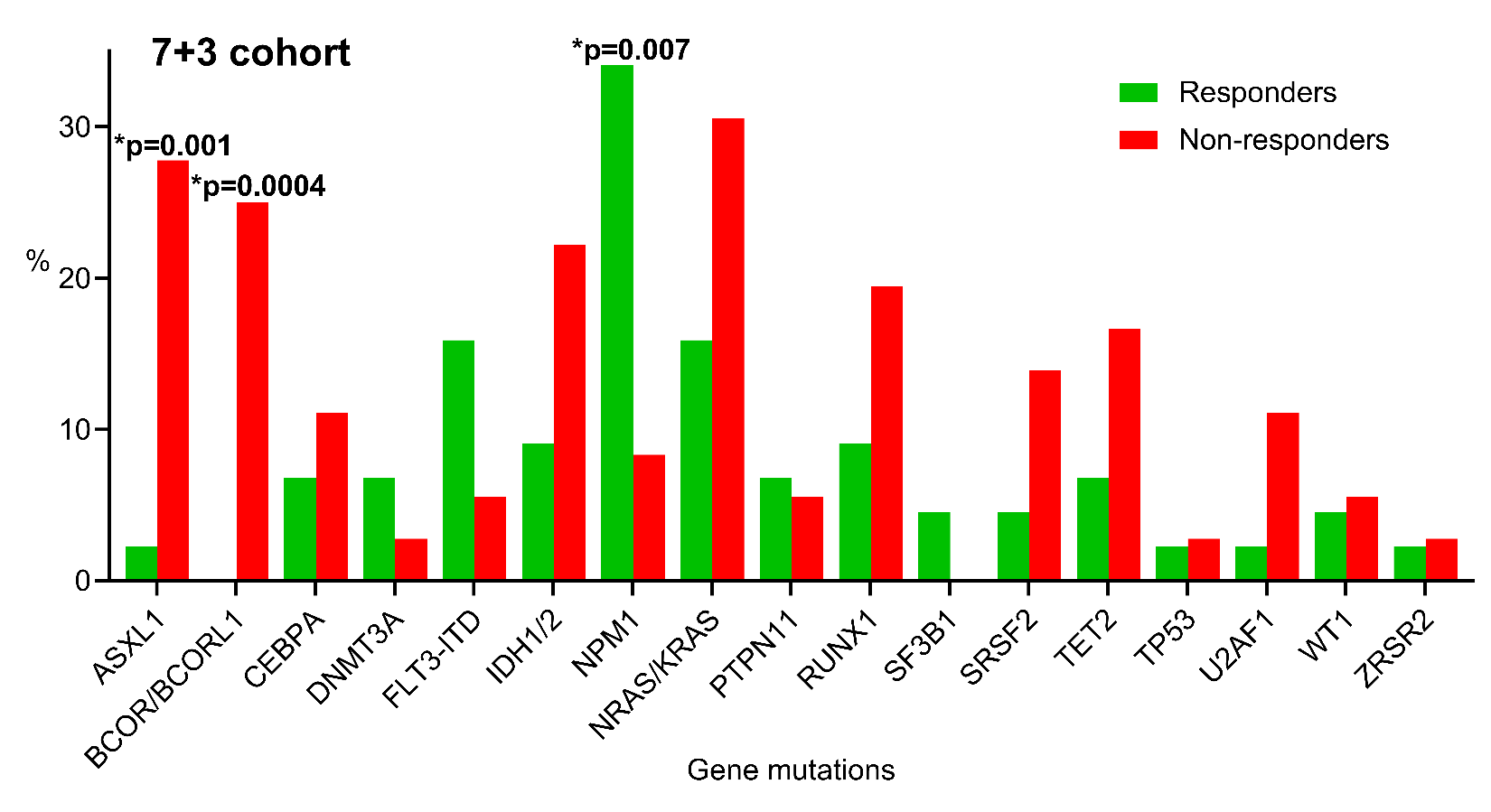


**Supplemental Figure 3: Molecular determinants of response to 7+3 treatment.**  Bar diagrams showing the percentages of gene mutations detected in responders vs non-responders to 7+3 regimen. All p- values were two-sided; those less than 0.05 were considered statistically significant.
